# Marine Communities on Oil Platforms in Gabon, West Africa: High Biodiversity Oases in a Low Biodiversity Environment

**DOI:** 10.1371/journal.pone.0103709

**Published:** 2014-08-01

**Authors:** Alan M. Friedlander, Enric Ballesteros, Michael Fay, Enric Sala

**Affiliations:** 1 Fisheries Ecology Research Laboratory, Department of Biology, University of Hawaii, Honolulu, Hawaii, United States of America; 2 Pristine Seas, National Geographic Society, Washington, DC, United States of America; 3 Centre d'Estudis Avançats-CSIC, Blanes, Spain; 4 Wildlife Conservation Society, Bronx, New York, United States of America; 5 Special Advisor, Presidence de la République, Libreville, République Gabonaise; U.S. Geological Survey, United States of America

## Abstract

The marine biodiversity of Gabon, West Africa has not been well studied and is largely unknown. Our examination of marine communities associated with oil platforms in Gabon is the first scientific investigation of these structures and highlights the unique ecosystems associated with them. A number of species previously unknown to Gabonese waters were recorded during our surveys on these platforms. Clear distinctions in benthic communities were observed between older, larger platforms in the north and newer platforms to the south or closer to shore. The former were dominated by a solitary cup coral, *Tubastraea* sp., whereas the latter were dominated by the barnacle *Megabalanus tintinnabulum*, but with more diverse benthic assemblages compared to the northerly platforms. Previous work documented the presence of limited zooxanthellated scleractinian corals on natural rocky substrate in Gabon but none were recorded on platforms. Total estimated fish biomass on these platforms exceeded one ton at some locations and was dominated by barracuda (*Sphyraena* spp.), jacks (Carangids), and rainbow runner (*Elagatis bipinnulata*). Thirty-four percent of fish species observed on these platforms are new records for Gabon and 6% are new to tropical West Africa. Fish assemblages closely associated with platforms had distinct amphi-Atlantic affinities and platforms likely extend the distribution of these species into coastal West Africa. At least one potential invasive species, the snowflake coral (*Carijoa riisei*), was observed on the platforms. Oil platforms may act as stepping stones, increasing regional biodiversity and production but they may also be vectors for invasive species. Gabon is a world leader in terrestrial conservation with a network of protected areas covering >10% of the country. Oil exploration and biodiversity conservation currently co-exist in terrestrial and freshwater ecosystems in Gabon. Efforts to increase marine protection in Gabon may benefit by including oil platforms in the marine protected area design process.

## Introduction

Gabon, West Africa, is situated in the Guinea Current Large Marine Ecosystem (GCLME), one of the world's most productive ocean regions, which is rich in fisheries resources, petroleum production, and is crucial to the lives of the region's 300 million coastal residents [1]. Despite this importance, the marine biodiversity of Gabon is poorly known owing to limited financial resources, lack of regional expertise, and a greater emphasis on extractive resources. Previous research has focused on the deep-sea biodiversity of the continental margin associated with oil and gas exploration [2], or related to continental shelf and slope fisheries resources [3]. The continental margins possess small methane-rich cold-see sites and associated non-biogenic carbonate reefs [1], [4], [5]. However, most of Gabon's shelf area is sandy or muddy, with limited hard bottom habitat. The dozens of oil platforms along the shelf may provide the only hard substrate for thousands of km^2^ [3].The marine life on these structures has never been studied and here we report on the first scientific surveys of Gabon's oil platforms.

The sea bottom of the Gulf of Guinea is characterized by terrigenous and sandy muds with low habitat and seascape diversity [6]. The Congo River, the second most voluminous river in the world after the Amazon, supplies a large amount of particulate and dissolved organic matter to the ocean, both at the surface and at the seafloor through the Congo Canyon and into the offshore waters of Gabon [7]. In addition, the Ogooué River in central Gabon has an average discharge rate of 4,758 m^3^ s^−1^, making it the third most important river in west equatorial Africa [8], [9]. Although the water discharge is one order of magnitude lower compared to the Congo River, the particulate load of the two rivers is similar [10]. Collectively these river systems greatly influence the marine ecosystem of Gabon.

The shelf off Gabon is dominated by sandy, sand-shell and gravel bottoms, becoming muddy toward the shelf [3]. Factors contributing to the enrichment in nutrients are related to seasonal up-welling, the discharge from the Congo and Ogooué rivers, and shelf-break upwelling [11]. A strong primary productivity gradient exists from north to south with the south showing higher productivity [12]. Nearshore waters off Gabon are generally warmer, with lower salinity and higher oxygen concentrations compared with more offshore environments [3]. These gradients are likely important to the distribution and abundance of marine organisms along the continental shelf of Gabon.

Ocean productivity in Gabon is dominated by seasonal equatorial upwelling and is strongly influenced by freshwater and sediments from the Congo and Ogooué rivers, particularly in nearshore waters [13]. The Benguela Current is a strong biogeographic barrier that prevents species from the southwestern region of the African continent and the Indian Ocean from colonizing the Gulf of Guinea [14], [15]. As a result of these oceanographic processes, the marine communities of the Gulf of Guinea are considered unique and a hotspot of marine biodiversity due primarily to the high proportion of endemic species [16]. Despite the high productivity of the region and its high degree of endemism, we know very little about Gabon's subtidal communities, with the exception of demersal fishes targeted by the large, industrial trawl fishery [3]. There are no true coral reefs in Gabon, and most of the largely unmapped reefs are rocky [17]. Except for a checklist of scleractinian corals for the region [17], there have been no studies of Gabon's subtidal reefs.

Extensive oil exploration and development began onshore in Gabon in the 1950s but offshore exploration did not begin until the 1960s [18]. There are currently >40 offshore oil platforms in Gabon yet virtually nothing is known about the marine biological communities associated with these platforms. Oil and gas platforms act as artificial reefs on continental shelves and provide hard substrate in open water that might otherwise be unavailable to marine organisms requiring such habitat [19], [20]. As a result of the limited reefs and the high biodiversity potential of the region, these platforms may provide a unique and important habitat for marine communities in Gabon.

Offshore oil and gas platforms are among the largest artificial structures in the ocean [21]. Worldwide there are >7,500 oil platforms [22], [23], and these structures are colonized by diverse ecological communities that have, in some instances, been shown to enhance biodiversity and fisheries production [24]–[26]. Approximately 40,000 oil and gas wells have been drilled in the northern Gulf of Mexico (GOM) since the 1940s [27]. There are currently about 3,600 oil and/or gas production platforms in the GOM and these platforms act as hard substratum upon which many reef organisms can settle and grow in a region where very little hard substratum now exists, nor has existed in recent geological time [27], [28]. Oil and gas platforms in southern California harbor diverse fish and invertebrate populations that are considered *de facto* artificial reefs and serve as important habitat for a number of species [29]–[31]. Off the north-western Australia continental shelf, a diverse range of taxa were observed on oil industry structures, including reef-dependent species and transient pelagics [23]. These platforms provide habitat that potentially increases the growth and survival of individuals, affording shelter for protection from predation and spawning substrate, and acting as a visual attractant for organisms not otherwise dependent on hard bottom, such as pelagic species [32]. However, hazards to shipping, exclusion of certain fisheries (e.g., trawling), spread of alien species, and potential spills and leaks have all been cited as some of the negative consequences associated with oil platforms, particularly older ones that are no longer in production [33]–[35].

The objectives of this study were to describe the marine communities on oil platforms in Gabon, determine the biogeographic affinities of the associated species, and discuss the implications of these platforms for marine conservation.

## Methods

The Government of Gabon granted all necessary permission to conduct this research. No vertebrate sampling was conducted and therefore no approval was required by the Institutional Animal Care and Use Committee.

### Oil platforms

**Figure 1:**
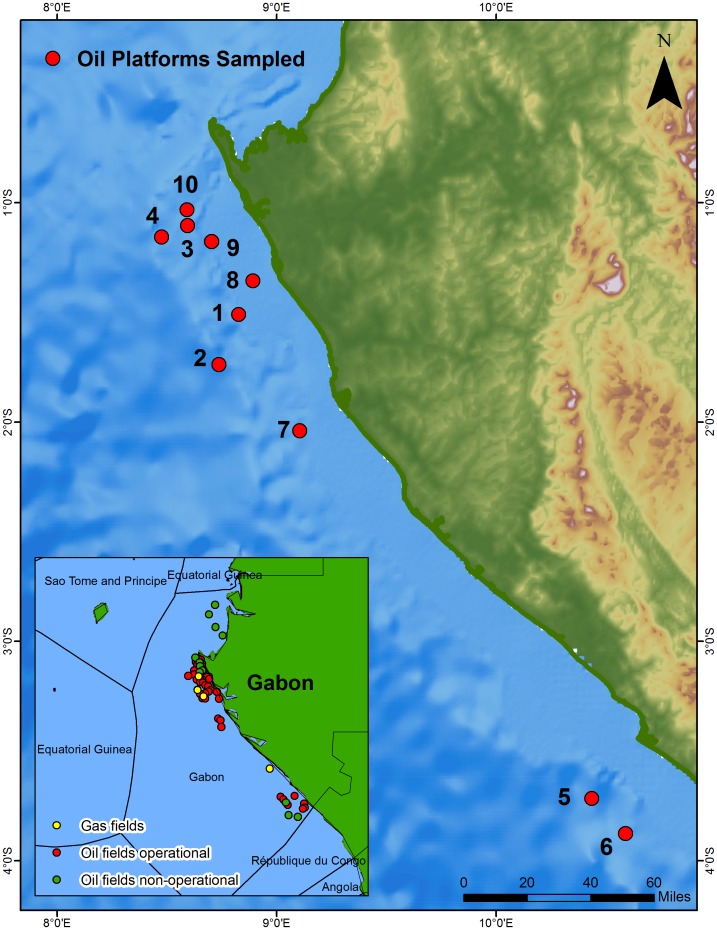
Oil platforms sampled along the coast of Gabon, West Africa. Platform numbers and descriptions are found in Table 1.

The oil platforms off Gabon investigated in this study sit on sandy and muddy bottoms at depths ranging from 26 to 102 m (Table 1, Fig. 1). The platforms had three to four vertical pillars linked by crossbeams at different depths. The shallowest crossbeams were at depths between 6–10 m, and the second shallowest (‘deep’ crossbeam hereafter) between 13–24 m (Table 1). There were a number of deeper crossbeams depending on the size and depth of the platform but these were beyond scuba depths and were not surveyed. We conducted quantitative surveys of benthic communities and fish assemblages along the two shallowest crossbeams at each of the 10 oil platforms surveyed. Since these crossbeams were not independent samples, values from benthic and fish surveys were averaged to derive density estimates for each platform.

**Table 1:** Descriptions of oil platforms sampled along the coast of Gabon, West Africa.

							Deep* crossbeam			Shallow beam			
Date	Platform no.	Company	Install year	Lat.	Long.	Bottom depth (m)	Depth (m)	Length (m)	Area (m^−2^)	Depth (m)	Length (m)	Area (m^−2^)	Flare
9-Oct-12	1	Total Gabon	1973	−1.509	8.827	43	19.0	16.0	64.0	6.0	13.0	52.0	Yes
9-Oct-12	2	Total Gabon	1980	−1.738	8.738	45	17.0	19.6	78.4	6.0	16.6	66.4	No
10-Oct-12	3	Total Gabon	1975	−1.103	8.594	62	19.0	18.3	73.0	6.0	15.0	60.0	No
10-Oct-12	4	Total Gabon	1974	−1.155	8.475	102	20.2	18.1	72.5	7.1	12.4	49.5	No
13-Oct-12	5	Vaalco	2001	−3.717	10.436	45	13.3	13.0	52.0	-	-	-	No
13-Oct-12	6	Vaalco	2001	−3.877	10.590	45	13.2	13.0	52.0	-	-	-	No
15-Oct-12	7	Perenco	1975	−2.039	9.105	45	23.8	6.0	24.0	6.7	9.0	36.0	No
18-Oct-12	8	Perenco	1975	−1.357	8.892	26	15.0	10.0	40.0	-	-	-	No
18-Oct-12	9	Perenco	1975	−1.177	8.703	31	20.0	5.0	78.5	10.0	5.0	78.5	No
18-Oct-12	10	Perenco	1975	−1.033	8.592	50	19.0	20.0	80.0	8.0	18.0	72.0	Yes

* ‘Deep’ is the second shallowest crossbeam of each platform.

### Benthic cover

Benthic cover was quantified *in situ* on the crossbeams using 0.25 m×0.25 m quadrats divided into 25 sub-quadrats of 5×5 cm [36], [37]. Eight quadrats were positioned haphazardly along every crossbeam, resulting in 24–32 quadrats for every depth and platform. Algae and other benthic cover (sponges, hydrozoans, soft and hard corals, barnacles, mollusks, echinoderms, and ascidians) within each quadrat were identified to the lowest possible taxonomic level. The percentage of sub-quadrats in which a species appeared was recorded and used as a measure of occurrence. A highly abundant taxon that appeared in all 25 subquadrats would produce a presence of 100%, whereas the total lack of a taxon would produce a presence of 0% [36]. The final abundance of a taxon within each depth and oil platform was then calculated as the mean of the percentage presence values of the quadrats sampled. The sum abundance of all quadrats exceeded 100% due to the combined presence of several taxa on the same sub-quadrat. Field visual survey methods did not allow identification of the smallest species (turf and filamentous algae, hydrozoans), which were sometimes pooled into groups during visual estimations. Vagile crustaceans (e.g. decapods) were not quantified in the quadrats, although the presence of lobsters during the survey was noted since they were conspicuous and commercially important.

### Fish assemblages

We conducted underwater visual fish censuses on oil platforms using two methods. First, we estimated the numerical density and size of individuals of each species along 4-m wide × 4-m tall belt transects along the ‘shallow’ crossbeams and the ‘deep’ crossbeams on each oil platform. Depth and length of transects varied corresponding to the length of the crossbeam where the transects were located (Table 1). Crossbeam lengths were measured upon completion of each quantitative survey. Because many of the schooling pelagic species (e.g., jacks, barracudas) were loosely associated with the platform structure, we estimated the numbers and sizes of these species that were visible during the dive to calculate their standing stock around each platform since they were underrepresented on quantitative transects. Although estimates associated with this method are constrained by schooling behavior, water clarity, and species identification, it complements the finer resolution belt transects by providing valuable information on highly vagile, and commercially important species that would have otherwise not been quantified.

Length estimates of fishes from both survey methods were converted to mass (M) using the following length–mass relationship: M =  aTL^b^, where the parameters a and b are constants for the allometric growth equation and TL is total length in cm [38]. From belt transects, all biomass estimates were converted to metric tons per hectare (t ha^−1^) and numerical abundance estimates were converted to number of individuals m^−2^. All fishes observed were categorized into four trophic groups (piscivores, carnivores, planktivores, and herbivores; [39], [40]), and classified into province and regional affinities based on [41].

### Statistical analysis

Non-metric multi-dimensional scaling (nMDS) analysis was used to examine differences in benthic communities and fish assemblages among oil platforms (PRIMER v.5 – [42]). Separate Bray–Curtis similarity matrices were created using abundance of benthic components at the lowest possible taxonomic level and fish numerical density (no. m^−2^). Prior to conducting the nMDS, benthic data were arcsin square root transformed, while fish density was square root transformed. The primary taxa vectors driving the ordination (Pearson correlation Product movement correlations ≥0.9 for benthos, ≥for 0.7 for fishes) were overlaid on the nMDS plot to visualize the major taxa that explained the spatial distribution patterns observed

Benthic taxon and fish species diversity were calculated from the Shannon-Weaver diversity index [43]: 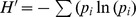, where *p_i_* is the proportion of all individuals counted that were of taxa *i*. Fish trophic biomass data did not conform to parametric statistical assumptions despite various transformations, therefore comparisons among trophic groups were conducted using a Kruskal-Wallis rank-sum test (H) with Dunn's test for unplanned multiple comparisons [44].

To describe the pattern of variation in community structure (patterns of distribution of biomass/abundance of high-level functional groups within the community) and their relationship to environmental-human gradients we used linear ordination methods. Linear models are appropriate for these data because a preliminary detrended correspondence analysis showed short gradient lengths (<2 SD) [45]. To explore the spatial distribution of community structure across oil platforms and its relationship with environmental variables (see below), we performed a direct gradient analysis (redundancy analysis: RDA) on the log-transformed community and environmental data matrices using the ordination program CANOCO for Windows version 4.0 [46]. The RDA introduces a series of explanatory (environmental) variables and resembles the model of multivariate multiple regression, allowing us to determine what linear combinations of these environmental variables determine the gradients. We pooled data from all taxa into the following community groups to facilitate the large-scale analysis: biomass of fish trophic groups (e.g., piscivores, carnivores, planktivores, and herbivores) and higher order benthic groups (e.g., Rhodophyta, Porifera, Cnidaria-Anthozoa, Cnidaria-Hydrozoa, Crustacea, Mollusca-Bivalvia).

The environmental data matrix included the following variables: latitude, longitude, depth, age of oil platform, platform area, distance offshore, and distance from the Ogooué and Congo river mouths. Correlations among environmental variables were examined using Spearman's rank-order correlation coefficients. Distance to the Congo and Ogooué rivers were significantly correlated with latitude and longitude (all p<0.05) and were excluded from the analysis. Longitude and depth, which were significantly correlated with latitude and platform area respectively (p<0.05), also were excluded from the analysis. To rank environmental variables in their importance for being associated with the structure of communities, we used a forward selection where the statistical significance of each variable was judged by a Monte-Carlo permutation test [47].

## Results

### Benthic composition

**Figure 2:**
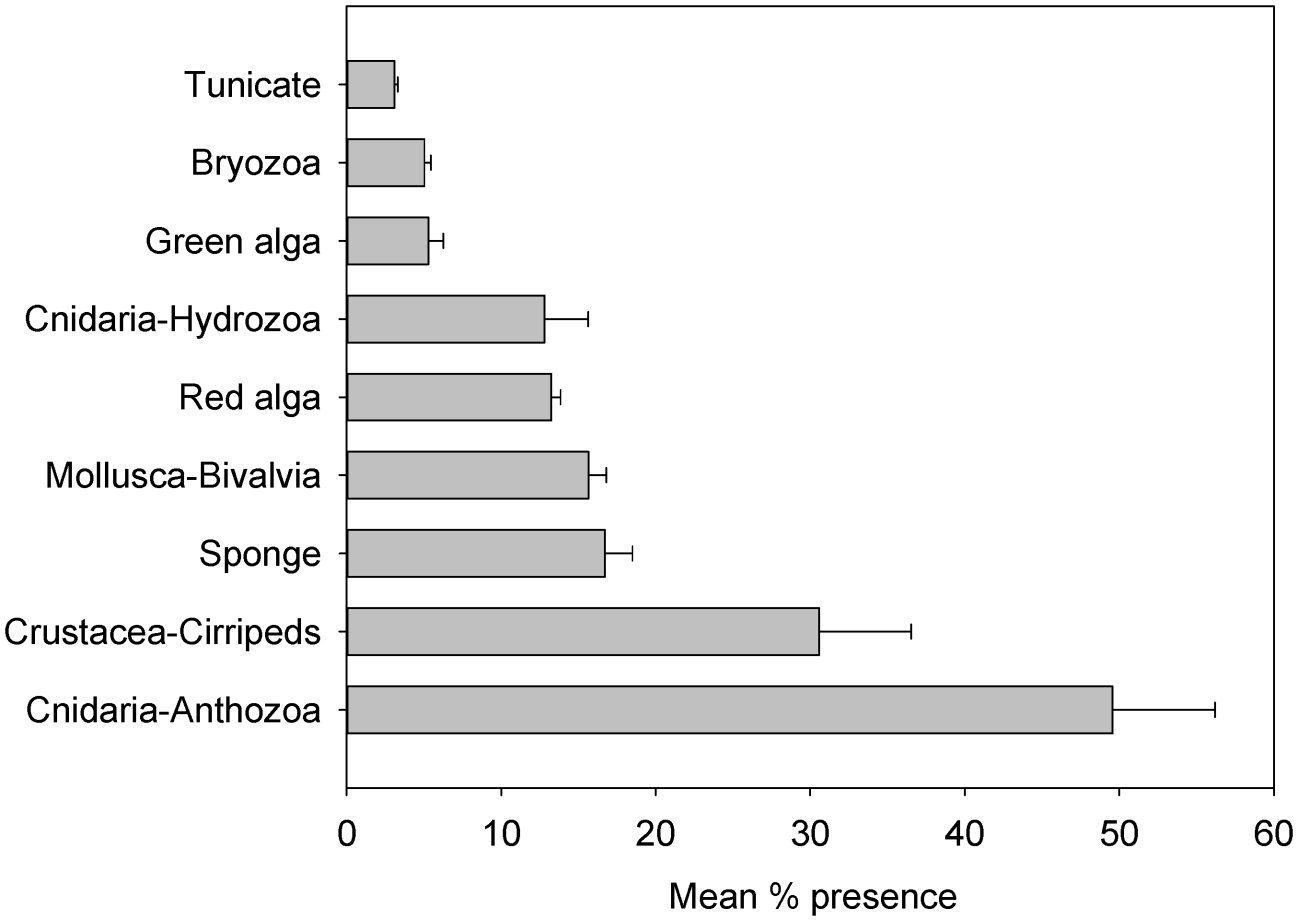
Benthic taxa on transects associated with oil platforms in Gabon, West Africa. Values are mean percent presence and standard error of the mean.

A total of 45 benthic taxa were recorded within quadrats on oil platforms sampled during our expedition (Table S1). Anthozoans made up 32.2% of the mean percent presence, followed by Cirriped crustaceans (19.9%), sponges (10.9%), bivalve mollusks (10.2%), and hydrozoans (8.3%). A solitary, undetermined cup coral (*Tubastraea* sp.) was the dominant species on many of the platforms, occurring on all platforms and accounting for 30.7% of the mean percent presence of all taxa (Table 2, Fig. 2). A barnacle, *Megabalanus tintinnabulum*, comprised 18.6% of the mean percent presence of all taxa and was present on 80% of the platforms. A sponge, *Haliclona* sp., accounted for 9.2% of the mean percent presence of all taxa, followed by an unidentified hydrozoan (9.2%), and two bivalve mollusks – *Crassostrea gassar* (7.6%) and *Dendostrea frons* (6.4%).

**Table 2:** Percent occurrence of major taxa found on oil platforms in Gabon.

Group	Taxa	Mean % (sd)	% freq.
Cnidaria-Anthozoa	*Tubastraea* sp.	47.34 (39.50)	100
Crustacea-Cirripedia	*Megabalanus tintinnabulum*	28.63 (33.33)	80
Sponge	*Haliclona* sp.	14.23 (15.61)	80
Cnidaria-Hydrozoa	*Hidrarians* unidentified	12.73 (16.29)	90
Mollusca-Bivalvia	*Crassostrea gasar*	7.61 (9.52)	100
Mollusca-Bivalvia	*Dendostrea frons*	6.36 (9.30)	60
Red alga	*Antithamnionella elegans*	6.15 (7.36)	60
Green alga	*Bryopsis plumosa*	5.25 (6.92)	50
Bryozoa	*Pentapora*-like	3.03 (5.10)	30
Red alga	Red algal turf	2.36 (5.15)	40

The mean is the percentage of the sub-quadrats were the taxa was observed. % freq. is the percentage of platforms upon which this taxon was observed (n = 10).

**Figure 3:**
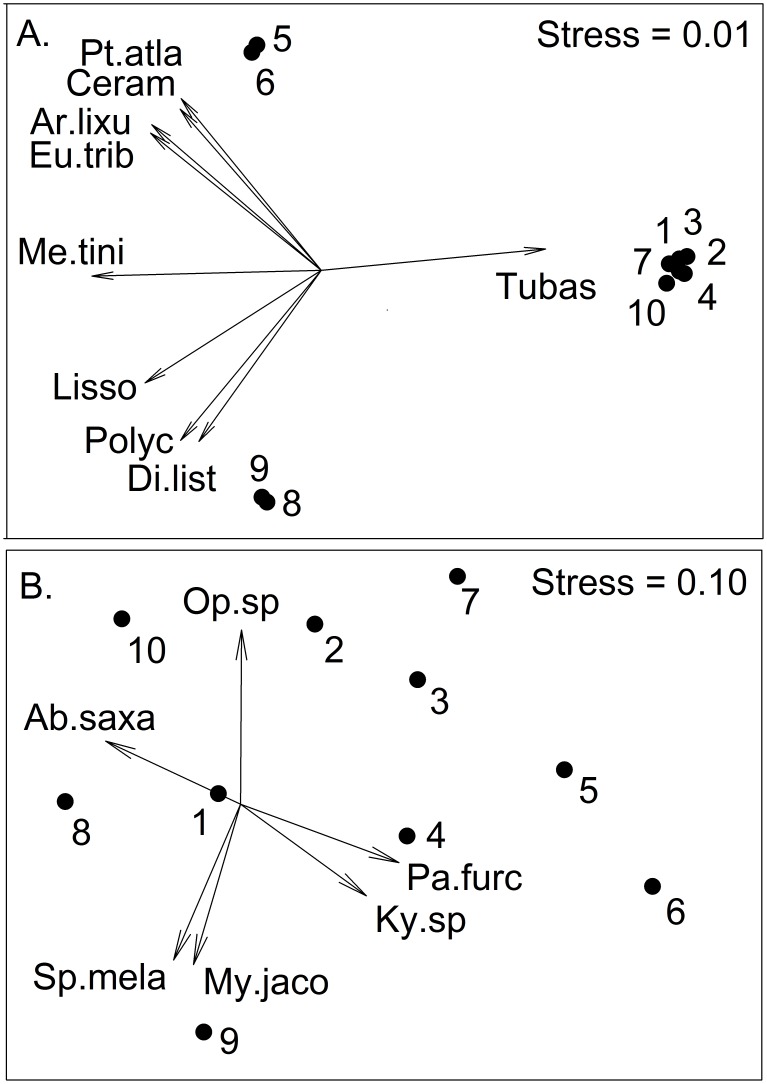
Non-metric multidimensional scaling plot of benthic communities and fish assemblages associated with oil platforms in Gabon. Vectors are the primary taxa driving the ordination (Pearson Product movement correlations ≥0.9). A. benthic communities species codes: Ar.lixu  =  *Arbacia lixula v. africana*, Ceram  =  *Ceramiaceae* unidentified, Di.list  =  *Diplosoma listerianum*, Eu.trib  =  *Eucidaris tribuloides v. africana*, Liss  =  *Lissoclinum* sp., Me.tini  =  *Megabalanus tintinnabulum*, Poly  =  Polyclinidae unidentified, Pt.atla  =  *Pteria atlantica*, Tubas  =  *Tubastraea* sp. B. Fish assemblage species codes: Ab.saxa  =  *Abudefduf saxatilis*, Ky.sp  =  *Kyphosus* sp., My.jaco  =  *Myripristis jacobus*, Op.sp  =  *Ophioblennius* sp., Pa.furc  =  *Paranthias furcifer* Sp.mela  =  *Spicara melanurus*.

Based on benthic community composition, the platforms formed three distinct clusters in ordination space (Fig. 3). The clusters dominated by *Tubastraea* sp. were highly concordant and well separated from the platforms to the south (5 and 6) and the two inshore platforms (8 and 9). These southern and inshore platforms were dominated by the barnacle *Megabalanus tintinnabulum*, but had much more diverse assemblages compared to the ones dominated by *Tubastraea* sp. The average number of taxa was highest on the inshore platforms (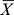 = 18.0±5.7) and lowest on the *Tubastraea* dominated ones (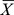 = 14.8±2.4). Diversity was highest on the southern platforms (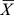 = 2.0±0.1) and lowest on the *Tubastraea* dominated platforms (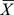 = 1.5±0.4).

Although not present on quadrats, we commonly observed two commercial species of lobsters on the platforms: spiny lobsters (*Panulirus regius*) and slipper lobsters (*Scyllarides herklotsii*). Spiny lobsters were found in groups of 2–4 individuals and were of small size (10–15 cm total body length), while slipper lobsters were often found alone or in pairs and attained larger adult sizes (20–25 cm total length). No estimates of lobsters by platform are provided as we did not use a specific protocol for quantifying these species.

### Fish assemblage characteristics

Based on all quantitative and qualitative surveys we observed a total of 65 fish species associated with oil platforms during this study (Table S2). Of these, 22 (34%) are new records for Gabon and 4 (6%) are new to the tropical West African coast. Assemblage richness consisted of 32% tropical eastern Atlantic (TEA) endemics and 31% amphi-Atlantic species (those found on both sides of the Atlantic), followed by circumtropical species (17%). Based on quantitative surveys, amphi-Atlantic species accounted for 73% of the numerical abundance, and 76% of the biomass observed on oil platforms along the coast of Gabon. Species endemic to the TEA only accounted for 21% of the numerical abundance and 16% of the biomass observed on quantitative surveys, while circumtropical species accounted for an additional 4% of the abundance and 2% of the biomass. Of the 65 species observed, only 38 (42%) were recorded on our quantitative surveys. Most of the species not observed on quantitative surveys were either pelagic or encountered at the bases of the platforms, associated with the seabed, and therefore not encountered on transects on the crossbeams.

The number of fish species on transects ranged from a low of 7 on platform 6 to a high of 22 on platform 1 (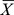 = 14.6±4.1). Higher numbers of species were observed on the platforms to the north, which were also the oldest platforms with the highest abundance of *Tubastraea* sp. The number of individuals observed on transects ranged from 0.9 m^−2^ on platform 2 to 8.5 m^−2^ on platform 10 (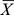 = 3.9±2.3), with no apparent pattern observed. Fish biomass among platforms also showed no clear pattern, with the highest biomass observed on platform 6 (10.8 t ha^−1^) and the lowest on platform 8 (0.9 t ha^−1^) with a mean of 4.3 (±3.3). Fish diversity varied without pattern and averaged 1.1 (±0.3) with the lowest diversity on platform 6 (H′ = 0.5) and highest on platform 9 (H′ = 1.6). Unlike the benthic communities, the fish assemblages associated with oil platforms did show spatial separation, with a small number of species accounting for much of the spatial variability (Fig. 3).

### Fish trophic structure

**Figure 4:**
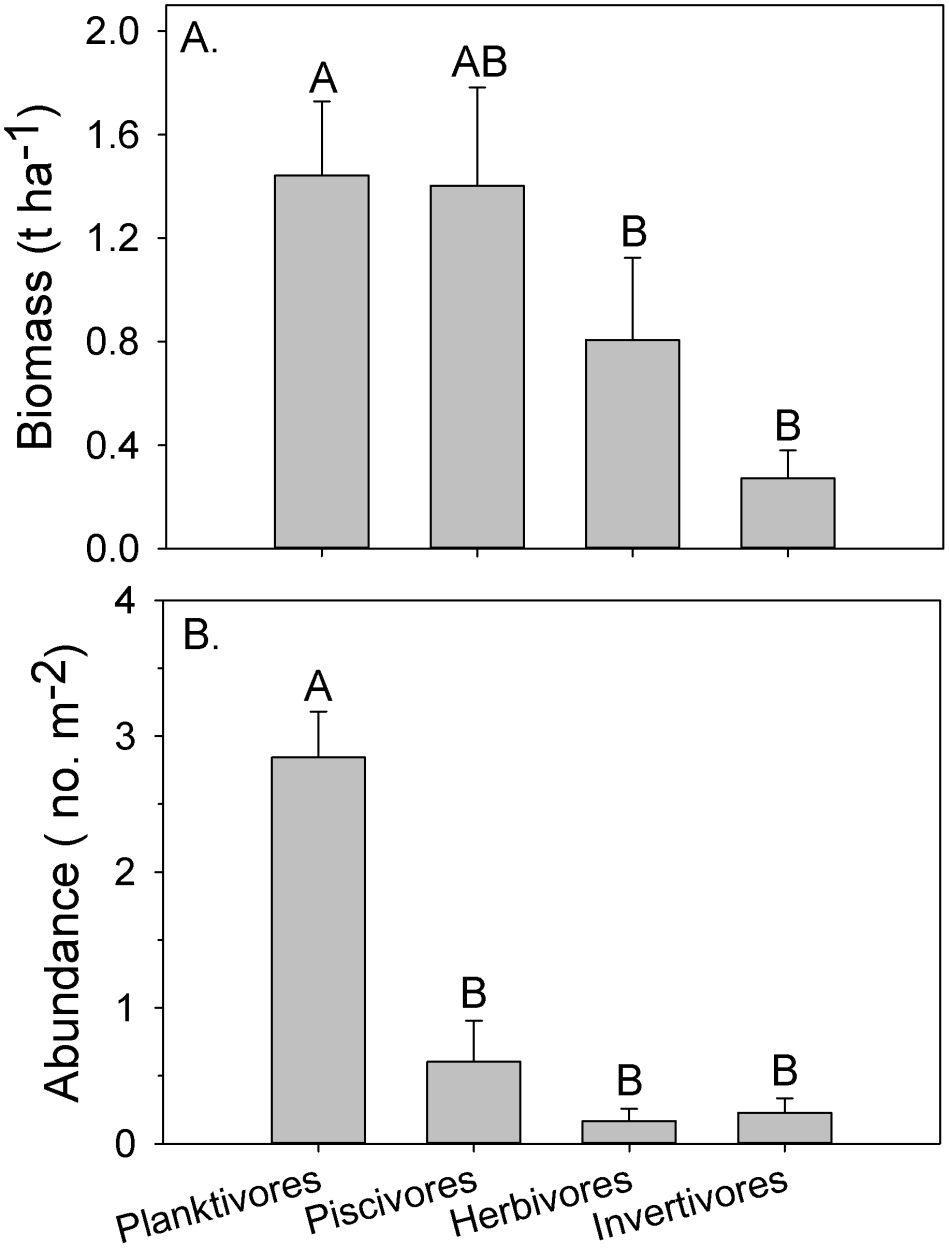
Comparisons of trophic (a) biomass (t ha^−1^) and (b) abundance (no. m^−2^) at oil platforms in Gabon. Values are means and standard error. Kruskal-Wallis Rank Sum comparisons among trophic groups were statistically different for biomass (H = 12.3, p = 0.007) and abundance (H = 35.3, p<0.001). Trophic groups with the same letter are not significantly different (Dunn's unplanned multiple comparisons procedures, α = 0.05).

Based on transect data, there were significant differences in the biomass and abundances among trophic groups observed on oil platforms (Fig. 4). Planktivores accounted for 74.0% of the numerical abundance and 36.8% of the overall biomass on platform transects. Planktivore abundance was highest at the two most southern platforms, which was also where the highest primary productivity occurs. Piscivores were the next most important trophic group by weight, accounting for 35.8% of the total biomass but 15.7% of the total abundance, with the highest biomass observed at the three north platforms (4, 9, and 10). Herbivores accounted for an additional 20.6% of the total biomass but only 4.3% of the numerical abundance. More than 80% of the total herbivore biomass was observed on the two most southern platforms. Invertivores accounted for 6.9% of the biomass and 5.9% of the abundance with no apparent pattern among platforms.

### Fish species composition

The creolefish, *Paranthias furcifer*, was the dominant fish species both numerically (32.2%) and by weight (26.3%) on oil platform transects (Table 3 and 4). The brown chromis, *Chromis multilineata*, was the second most important species numerically (27.1%) but only accounted for 3.4% of the total biomass. This species was followed in abundance by the African sergeant, *Abudefduf hoefleri*, comprising 13.4% of the total assemblage by numbers and 7.2% by weight. The second most important species by weight was the Bermuda chub, *Kyphosus sectator*, accounting for 23.5% of the total biomass, followed by blue runner, *Caranx crysos* (14.9%).

**Table 3:** Biomass (g m^−2^) of the top ten species observed on quantitative transects on oil platforms in Gabon, West Africa.

Family	Species	Common name	Distribution	Trophic	Mean (sd)	%
Groupers	*Paranthias furcifer*	Creolefish	Atlantic	Plank	112.7 (149.3)	26.3
Chubs	*Kyphosus sp*	Bermuda Chub	Atlantic	Herb	101.0 (213.5)	23.5
Jacks	*Caranx crysos*	Blue Runner	Atlantic	Pisc	63.8 (127.7)	14.9
Damselfishes	*Abudefduf hoefleri*	African Sergeant	West Africa	Plank	30.8 (32.5)	7.2
Snappers	*Lutjanus fulgens*	Golden African Snapper	West Africa	Pisc	16.2 (48.6)	3.8
Damselfishes	*Chromis multilineata*	Brown Chromis	Atlantic	Plank	14.4 (14.8)	3.4
Surgeonfishes	*Acanthurus monroviae*	Monrovia Doctorfish	Atlantic	Herb	13.9 (14.9)	3.2
Barracuda	*Sphyraena barracuda*	Great Barracuda	Circumtropical	Pisc	11.1 (15.3)	2.6
Jacks	*Caranx fischeri*	Longfin Crevalle Jack	West Africa	Pisc	10.8 (12.4)	2.5
Hawkfishes	*Cirrhitus atlanticus*	West African Hawkfish	West Africa	Invert	7.4 (7.6)	1.7

Values are means and one standard deviation. Trophic groups – Plank  =  planktivores, Herb  =  herbivores, Pisc  =  piscivores, Invert  =  invertivores. Distributions derived from [82], [83]. % is the percent contribution of each species to total fish biomass.

**Table 4:** Numerical abundance (no. m-2) of the top ten species observed on quantitative transects on oil platforms in Gabon, West Africa.

Family	Species	Common Name	Distribution	Trophic	Mean (sd)	%
Groupers	*Paranthias furcifer*	Creolefish	Atlantic	Plank	1.25 (1.68)	32.2
Damselfishes	*Chromis multilineata*	Brown Chromis	Atlantic	Plank	1.05 (1.13)	27.1
Damselfishes	*Abudefduf hoefleri*	African Sergeant	West Africa	Plank	0.52 (0.55)	13.4
Jacks	*Caranx* juveniles	Jacks	Atlantic	Pisc	0.31 (0.99)	8.0
Damselfishes	*Abudefduf saxatilis*	Sergeant-Major	Atlantic	Plank	0.14 (0.18)	3.6
Snappers	*Lutjanus fulgens*	Golden African Snapper	West Africa	Pisc	0.11 (0.34)	2.8
Chubs	*Kyphosus sp*	Bermuda Chub	Atlantic	Herb	0.10 (0.18)	2.6
Jacks	*Caranx crysos*	Blue Runner	Atlantic	Pisc	0.07 (0.14)	1.8
Damselfishes	*Stegastes imbricatus*	Cape Verde Gregory	West Africa	Herb	0.06 (0.08)	1.5
Wrasses	*Thalassoma newtoni*	Newton Wrasse	West Africa	Invert	0.06 (0.09)	1.5

Values are means and one standard deviation. Trophic groups – Plank  =  planktivores, Herb  =  herbivores, Pisc  =  piscivores, Invert  =  invertivores. Distributions derived from [82], [83]. % is the percent contribution of each species to total fish abundance.

### Associated fish assemblage

Since many of the vagile pelagic schooling species were not recorded on transects, we estimated total abundance and sizes of these species over the course of the dive that were associated with each oil platform. Nine species, primarily jacks and barracuda, made up the vast majority of schooling pelagic species associated with these platforms (Table 5). Estimates of standing stock were based on the estimated numbers in each school and the median size of individuals in the school. The standing stock of these pelagic species averaged 0.83 t (±0.24) per platform with a minimum of 0.57 tonnes on platform 8 and a maximum of 1.18 tonnes on platform 1. Great barracuda (*Sphyraena barracuda*) accounted for 33.2% of the average standing stock, followed by longfin jack crevalle (*Caranx fischeri* - 33.0%), and rainbow runner (*Elagatis bipinnulata* - 12.7%)

**Table 5:** Estimates of abundance and sizes of large schooling resource species associated with oil rigs in Gabon.

Species	Platform 1	Platform 2	Platform 3	Platform 4	Platform 5	Platform 6	Platform 7	Platform 8	Platform 9	Platform 10
*Caranx bartholomaei*										100
Yellow Jack										(40–45)
*Caranx crysos*					100	100				300
Blue Runner					(40)	(40)				(40–45)
*Caranx fischeri*	200	200	100	100	75	75	100	75	75	
Longfin Crevalle Jack	(50–60)	(50–60)	(50–60)	(50–60)	(50–75)	(50–75)	(60–75)	(65–70)	(65–75)	
*Caranx hippos*			50	50			75			
Crevalle Jack			(50–65)	(50–65)			(60–70)			
*Caranx latus*			50							
Horse-eye Jack			(60–65)							
*Elagatis bipinnulata*	100	100	50	75	50	50	75			150
Rainbow Runner	(65–70)	(65–70)	(65–75)	(65–75)	(75)	(75)	(65–80)			(70–80)
*Rachycentron canadum*								2		
Cobia								(120–130)		
*Sphyraena barracuda*	200	200	100	100	50	50		100	100	100
Great Barracuda	(80–100)	(80–100)	(75–100)	(75–100)	(75–90)	(75–90)		(80–100)	(80–100)	(75–100)
*Trachinotus ovatus*	50		25		75	75				
Pompano	(45–50)		(45–50)		(50–65)	(50–65)				

Values are approximate number of individuals associated with the oil rig with minimum and maximum size (TL [total length] cm) in parentheses.

### Fish spawning and courtship

On October 12, 2012, at 3 pm local time, we observed pair spawning of red snapper, *Lutjanus dentatus*, on platform no. 4 off Cape Lopez. A male (∼70 cm) and a female (∼50 cm) swam together from deeper water towards the surface, forming a spiral, and swimming apart while releasing eggs and sperm at 10 m depth. We observed two consecutive spawning events; the first outside of the platform structure, and the second within the structure. Immediately after spawning, dozens of creolefish swam towards the spawn cloud, to eat the eggs. We also observed the courtship behavior of the yellow jack, *Caranx bartholomaei*, at several platforms. Yellow jacks formed schools of up to several hundred individuals, most with typical silver body coloration. In the afternoon, a small number of individuals developed a dark coloration, and silver-dark pairs formed and swam together away from the school. Although we did not observe spawning, we believe this is courtship behavior that has been identified for other species of jacks (e.g., [48]).

### Community structure

**Figure 5:**
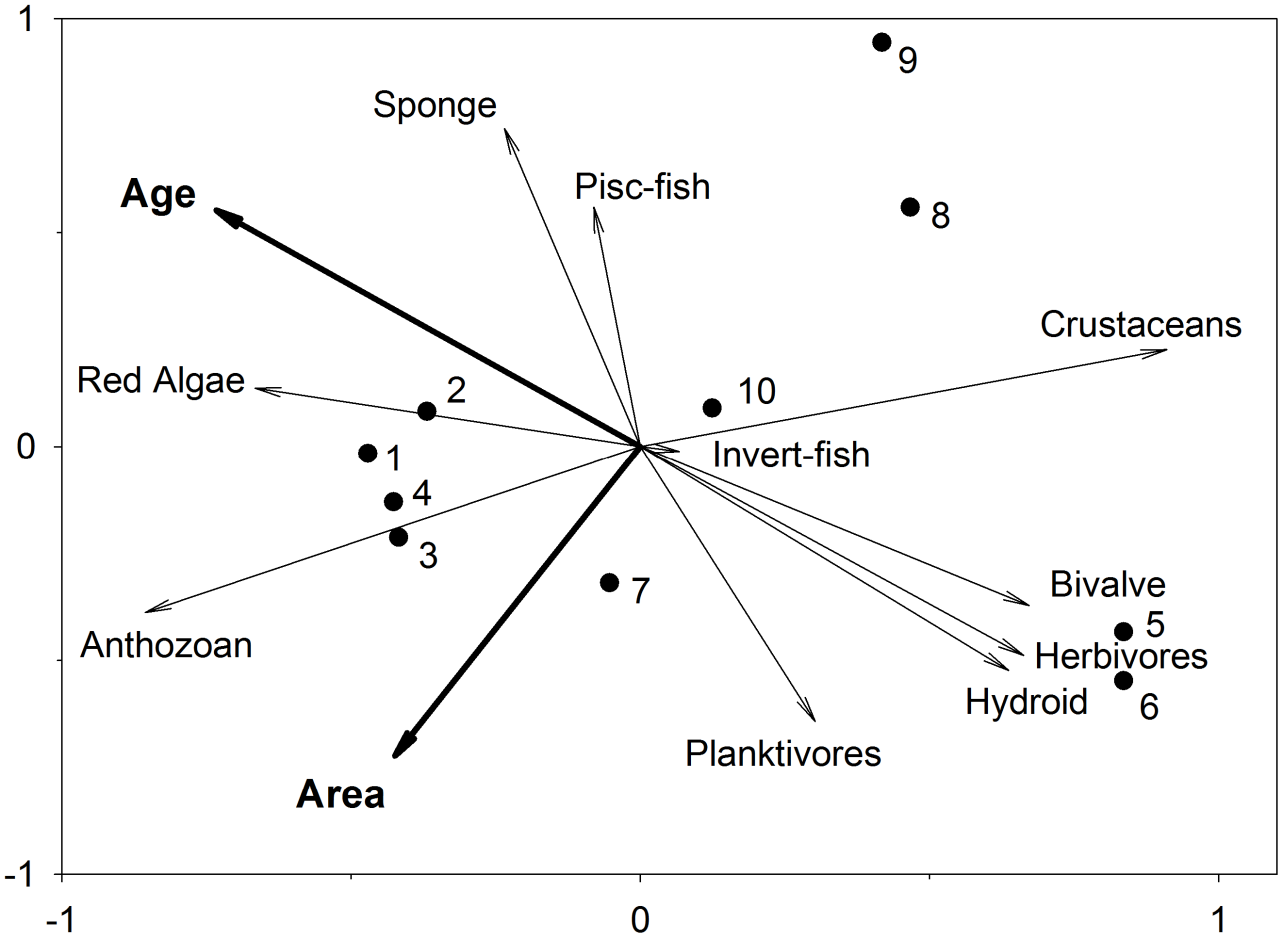
Biplot of results of redundancy analysis on biological (biomass of fish trophic groups and higher order benthic groups [e.g, Red alga, Sponge, Cnidaria-Anthozoa, Cnidaria-Hydrozoa, Crustacea-Cirripedia, Mollusca-Bivalve) and environmental data (e.g., age of platform, platform area).

Ordination of biomass/abundance of functional groups among oil platform communities revealed three groups of sites mainly consisting of: (1) platforms dominated by anthozoans and red algae (2) those dominated by piscivorous feeding fishes and sponges, and (3) platforms dominated by planktivorous and herbivorous fishes along with hydroids and bivalves (Fig. 5). The first two axes of the RDA biplot explained 78% of the functional group variance and 97% of the functional group-environment relationship (Table 6). The main factors involved in this ordination are platform age and area, which are orthogonal to one another in ordination space (Fig. 5, Table 7). Platform age and latitude showed similar ordination trajectories, reflecting the history of expansion of oil exploration in Gabon starting in Port Gentil and moving south to the border with Congo. Platform area is correlated with water depth and distance offshore, with assemblages becoming more diverse with increasing platform area, water depth, and distance from shore.

**Table 6:** Results of redundancy analysis (RDA) on log-transformed data on fish biomass of trophic groups and abundance of higher-level benthic groups with environmental variables (e.g., age of platform, distance offshore, latitude, platform area).

Axes	1	2	3	Total variance
Eigenvalues	0.59	0.19	0.01	1
FG-environment correlations	0.96	0.97	0.56	
Cumulative percentage variance				
of FG data	58.92	77.93	79.15	
of FG-environment relation	73.74	97.32	98.87	
Sum of all eigenvalues				1
Sum of all canonical eigenvalues				0.80

FG  =  functional groups.

**Table 7:** Conditional effects of Monte-Carlo permutation results on the redundancy analysis (RDA).

Variable	Lambda A	P	F
**Age**	0.40	**0.01**	5.4
**Area**	0.25	**0.01**	5.0
Offshore	0.09	0.13	1.9
Lattitude	0.06	0.17	1.6

Variables in bold are significantly correlated with the RDA axes.

## Discussion

This work represents the first scientific surveys of the marine ecosystems on oil platforms off West Africa. We found an extraordinary diversity and biomass of fishes and rocky invertebrates on these platforms that are in sharp contrast to much of Gabon's marine environment, which is dominated by soft sediment communities. Quantitative transect surveys were most useful in describing the biodiversity and abundance of “resident” fishes and benthic communities on oil platforms but they underestimate the standing stock of pelagic species that are not as closely associated with the platforms as other species. The estimated fish biomass on oil platforms exceeded 1 ton and was dominated by pelagic species (barracuda, rainbow runner, jacks). Large snappers (primarily *L. dentatus*) were often deeper and not easily observed, so the actual standing stock of fish biomass on these platforms is likely much higher than our estimates suggest. Elsewhere, the potential production around platforms is high as indicated by estimates of biomass of cod (*Gadus morhua*) and saithe (*Pollachius virens*) around oil platforms in the North Sea, which range from 7–12 tonnes [49].

Owing to the known prevalence of resource species on these platforms, spear fishermen frequent them, particularly out-of-service ones near the population centers of Libreville and Port Gentil. Videos and anecdotal information suggest substantial hunting of goliath grouper (*Epinephelus itajara*), barracuda, cobia (*Rachycentron canadum*), Guinean pompano (*Trachinotus maxillosus*), cubera snapper (*Lutjanus agennes*), jacks (Carangidae) and several species of carcharhinid sharks. Despite this fishing pressure, fish biomass at some platforms is larger than is reported in the literature for most tropical reef fishes, and even higher than many pristine reefs surveyed in the Pacific [50]–[52]. This high standing stock of fishes is possible because of the high productivity of the region, and because many of the platforms are largely unfished since they are de facto MPAs owing to security restrictions. As on pristine reefs, top predators account for a large part of the fish biomass. Before the systematic spearfishing of the large groupers, snapper, jacks, and sharks, biomass and dominance of predators must have been even higher.

Most of the fish biomass on the platforms was composed of pelagic species with broad biogeographic distributions. However, much of the observed species richness consisted of demersal species, many of which had distinct and unique assemblages. Studies at nearby São Tomé and Príncipe found the archipelago fish fauna to consist of both western and eastern Atlantic species and noted that the easterly flowing Equatorial currents (the seasonal Equatorial Counter Currents and the subsurface Equatorial Undercurrent) link the western Atlantic and the eastern Atlantic at this latitude [53], [54]. Likewise, the marine invertebrate fauna of São Tomé and Príncipe is known to consist of a mix of the two faunal regions [17], [55]–[59]. Oil platforms off Gabon may therefore help extend this unique assemblage onto the West African shelf.

Despite its proximity to the African continent, these platforms harbor a unique fish fauna, with a large proportion of the assemblage composed of amphi-Atlantic species. Several amphi-atlantic species, which, in the Eastern Atlantic occur only around oceanic islands (e.g., *Epinephelus adscensionis, Paranthias furcifer, Mulloidychtis martinicus, Bodianus pulchellus, Chromis multilineata, Gnatholepis thomsoni, Melychthis niger*, [53]) were observed on the oil platforms off Gabon. The wrasse *Thalassoma newtoni* was considered endemic to the islands of São Tomé but has recently been recorded from Senegal [60]. We observed this species on all of the platforms surveyed, where it was very common. These facts highlight how these platforms act like small islands in an otherwise featureless seascape across the continental shelf of Gabon. We observed spawning and spawning behavior for two commercially important snappers on the platforms, and they are likely spawning sites for numerous other species as well. Therefore, these platforms are likely sources for the replenishment of other platforms and the scarce reefs in the region, acting like distinct populations in a metapopulation [61]. They could be of great value to fisheries conservation for commercially harvested fish species that associated with these platforms.

Some species of invertebrates from our surveys have not been successfully identified to the species level and taxonomists are currently working on them. Although several species of scleractinian corals have been reported from Gabon [10], no true coral reefs are found. Despite the fact that oil platforms provide suitable substrate for the settlement of corals in other locations, no hermatypic corals were recorded on the platforms during our surveys. The ahermatypic dendrophyllid *Tubastraea* is circumtropical in distribution but likely a relatively recent invader to the Gulf of Guinea, possibly coming from the Indo-Pacific via the Panamenian region (Óscar Ocaña, pers. comm.). The taxonomy of the genus *Tubastraea* is currently under debate.

### Conservation implications

Artificial reefs are increasingly being applied in mitigation efforts for natural systems – including their use as physical barriers to discourage illegal trawling in seagrass beds in Western Europe [62]. Oil platforms have been described as “de facto marine protected areas” [63], because they exclude trawl fishing and their large internal spaces offer shelter to fishes and other organisms. Platforms are complex structures, involving numerous crossbeams and large interstitial spaces, which increase overall habitat complexity and likely support high reef fish diversity and abundance.

The presence of oil platforms in the marine environment can create a number of environmental and social problems. The sinking of the Deepwater Horizon drilling rig created the largest marine oil spill in history (nearly 5 million barrels) and resulted in significant ecological and economic damage to the GOM region [64], [65]. Smaller spills and leaks from offshore platforms commonly occur around the world, and as oil exploration and extraction moves into ever deeper water and into stormier and icier seas, potential risks will increase [66], [67]. Invasive and exotic species have been reported on oil platforms around the world, where they compete with native species for space [20], [28], [68]. This is also an issue with decommissioned platforms that may be moved to a distant locations and enhance the spread of these exotic and invasive species [69]. Offshore platforms can also be a source of chronic stress that can lead to sub-lethal impacts on resident benthic organisms, resulting in loss of biodiversity [70].

Although often controversial, rigs-to-reef programs that allow decommissioned oil platforms to stay in place have gained some support by governmental and non-governmental organizations [71]. They are particularly effective when they serve as no-take reserves and included in a marine protected area network [72], [73]. Preliminary evidence indicates that decommissioned rigs in shallower waters can help rebuild declining fish stocks and therefore have high conservation value. Love and colleagues provided data that support the beneficial role of rigs for southern California's bocaccio rockfish (*Sebastes paucispinis*) populations [74]. They estimated that, of the average number of juveniles that survive annually across the geographic range of the species, approximately 20% (equivalent to ∼430,000 individuals) were supported by eight southern Californian rigs.

Population persistence in the deep sea relies heavily on connectivity between deep-sea communities on isolated reefs [75]. Over large distances, small, isolated reefs may act as stepping stones within an inhospitable matrix of soft sediment [19]. Propagules that settle on to artificial reefs in locations relatively isolated from natural reefs would likely not have found suitable natural habitat before perishing. In this case, artificial reefs could potentially increase biomass production by increasing settlement [19].

The addition of artificial reefs in the deep sea is likely to increase ecological connectivity, which will have important biogeographical consequences [19]. In the Gulf of Mexico, where the amount of natural hard substratum is limited, oil platforms contribute substantially to local and regional abundance of reef habitat and the abundance of reef-associated fishes [76]–[80]. Platforms have been shown to facilitate the expansion of coral populations in the Gulf of Mexico and therefore they possess an intrinsic environmental value through the presence of coral populations [20]. However it has also been shown that these platforms can act as vectors for invasive species.

Rocky bottoms are extremely scarce on the continental shelf of Gabon [3]. The addition of artificial reefs may contribute substantially to local and regional abundance of hard substrate habitat and the abundance of reef-associated fishes [76]–[80] and is likely to increase ecological connectivity [75]. Moreover, if fish populations are limited by the amount of available habitat, then the addition of suitable artificial habitat increases the environmental carrying capacity, resulting in a sustained increase in population biomass (the “production” hypothesis; [17]). Polovina and Sakai [81] showed that regional catch per unit effort of the Pacific giant octopus (*Octopus dofleini*) increased after the addition of artificial reefs in northern Japanese waters, and therefore supporting fisheries production. However, fish observed on artificial reefs may simply have been attracted to those locations from surrounding habitat (the “attraction” hypothesis; [17]). Our results suggest that these platforms increase local production through enhanced settlement, increased reproductive output, and likely through reduced natural and fishing mortality.

The Government of Gabon made history in 2002 when they placed nearly 11% of their land area under permanent protection in 13 National Parks. To date three marine protected areas have been designated within this region (Akanda National Park, Mayumba National Park, and Pongara National Park) and are located in inshore territorial and coastal waters, covering ∼1,745 km^2^; of these only Mayumba National Park is entirely a no-take MPA, whereas Akanda and Pongara National Parks are terrestrial in nature and have marine buffer components. In February 2014, President Ali Bongo Ondimba announced the creation of a system of Marine National Parks covering 20% of Gabon's exclusive economic zone. Between oil exclusion zones and Marine National Parks all of the oil infrastructure as of 2014 will be in no-take zones. These platforms should become core areas for replenishment of the marine parks and some of Gabon's fisheries.

## Supporting Information

Table S1 **List of macroalgae and invertebrates observed on oil platforms in Gabon.**
(DOCX)

Table S2 **List of fish species observed on oil platforms in Gabon.** Species ordered phylogenetically based on Eschmeyer 2013. Provinces based on [51], [66]–[67], NWA  =  Northwestern Atlantic, SWA  =  Southwestern Atlantic, MAR  =  Mid-Atlantic Ridge, NEA  =  Northeast Atlantic, TEA  =  Tropical Eastern Atlantic. New  =  new record for Gabon.
(DOCX)
