# Supplementary material for: Marine Communities on Oil Platforms in Gabon, West Africa: High Biodiversity Oases in a Low Biodiversity Environment
Source: PLoS One. 2014 Aug 1;9(8):e103709. doi: 10.1371/journal.pone.0103709 (PMC4118950; doi:10.1371/journal.pone.0103709)
Supplement: Table S1 — List of macroalgae and invertebrates observed on oil platforms in Gabon. (DOCX) [file pone.0103709.s001.docx]

Table S1. List of macroalgae and invertebrates observed on oil platforms in Gabon.

| Higher taxa | Taxa | Average | stdev | Sum of %_freq |
| --- | --- | --- | --- | --- |
| Brown alga | *Sargassum filipendula* | 0.04 | 0.14 | 100% |
| Red alga | *Antithamnionella elegans* | 6.15 | 7.36 | 30% |
| Red alga | *Ceramiaceae* unidentified | 0.78 | 1.92 | 10% |
| Red alga | *Dipterosiphonia?* | 0.01 | 0.04 | 40% |
| Red alga | *Encrusting coralline* | 0.37 | 1.17 | 10% |
| Red alga | *Gelidiopsis planicaulis* | 0.58 | 1.13 | 30% |
| Red alga | *Hypnea spinella* | 1.33 | 2.49 | 20% |
| Red alga | *Polysiphonia* sp. | 0.03 | 0.08 | 10% |
| Red alga | red algal turf | 2.36 | 5.10 | 40% |
| Red alga | *Rhodymenia pseudopalmata* | 1.64 | 5.18 | 10% |
| Green alga | *Bryopsis pennata* | 0.01 | 0.02 | 50% |
| Green alga | *Bryopsis plumosa* | 5.25 | 6.92 | 70% |
| Green alga | *Derbesia tenuissima* | 0.04 | 0.08 | 30% |
| Sponge | *Batzella sp.?* | 0.13 | 0.21 | 10% |
| Sponge | *Cliona sp.* | 0.01 | 0.04 | 10% |
| Sponge | *Haliclona sp.* | 14.23 | 15.61 | 30% |
| Sponge | Other sponges | 1.24 | 1.42 | 10% |
| Sponge | Red sponge encrusting | 1.09 | 2.17 | 10% |
| Sponge | *Scopalina sp.?* | 0.01 | 0.04 | 10% |
| Cnidaria-Anthozoa | *Bunodosoma* sp. | 0.04 | 0.12 | 100% |
| Cnidaria-Anthozoa | *Carijoa riisei* | 1.93 | 2.09 | 60% |
| Cnidaria-Anthozoa | *Diadume leucomela* | 0.24 | 0.75 | 60% |
| Cnidaria-Anthozoa | *Epizoanthus* sp. | 0.01 | 0.04 | 50% |
| Cnidaria-Anthozoa | *Tubastraea* sp. | 47.34 | 39.50 | 30% |
| Cnidaria-Hydrozoa | *Hidrarians unidentified* | 12.73 | 16.29 | 40% |
| Cnidaria-Hydrozoa | *Plumaria* sp. | 0.06 | 0.20 | 30% |

Appendix I. Continued.

| Higher taxa | Taxa | Average | stdev | Sum of %_freq |
| --- | --- | --- | --- | --- |
| Bryozoa | *Pentapora-*like | 3.03 | 5.10 | 80% |
| Bryozoa | *Schizoporella errata* | 1.60 | 3.78 | 90% |
| Polychaeta | *Salmacina* sp. | 0.70 | 0.84 | 40% |
| Mollusca-Bivalvia | *Crassostrea gasar* | 7.61 | 9.52 | 30% |
| Mollusca-Bivalvia | *Dendostrea frons* | 6.36 | 9.30 | 20% |
| Mollusca-Bivalvia | *Pinna rudis* | 0.14 | 0.29 | 60% |
| Mollusca-Bivalvia | *Pteria atlantica* | 1.53 | 3.35 | 30% |
| Mollusca-Gastropoda | *Stramonites haemastoma* | 0.14 | 0.43 | 20% |
| Crustacea | Cirripedia unidentified | 1.94 | 4.80 | 90% |
| Crustacea-Cirripedia | *Megabalanus tintinnabulum* | 28.63 | 33.33 | 20% |
| Echinoidea | *Arbacia lixula v. africana* | 0.85 | 1.75 | 10% |
| Echinoidea | *Diadema africanum* | 0.01 | 0.04 | 20% |
| Echinoidea | *Eucidaris tribuloides v. africana* | 0.34 | 0.66 | 30% |
| Tunicate | *Botrylloides leachii* | 1.72 | 5.37 | 10% |
| Tunicate | *Diplosoma listerianum* | 0.60 | 1.35 | 10% |
| Tunicate | *Lissoclinum* sp. | 0.63 | 1.22 | 10% |
| Tunicate | *Polyclinidae unidentified* | 0.14 | 0.36 | 10% |
