# Supplementary material for: Marine Communities on Oil Platforms in Gabon, West Africa: High Biodiversity Oases in a Low Biodiversity Environment
Source: PLoS One. 2014 Aug 1;9(8):e103709. doi: 10.1371/journal.pone.0103709 (PMC4118950; doi:10.1371/journal.pone.0103709)
Supplement: Table S2 — List of fish species observed on oil platforms in Gabon. Species ordered phylogenetically based on Eschmeyer 2013. Provinces based on [51], [66]–[67], NWA = Northwestern Atlantic, SWA = Southwestern Atlantic, MAR = Mid-Atlantic Ridge, NEA = Northeast Atlantic, TEA = Tropical Eastern Atlantic. New = new record for Gabon. (DOCX) [file pone.0103709.s002.docx]

Table S2. List of fish species observed on oil platforms in Gabon. Species ordered phylogenetically based on Eschmeyer 2013. Provinces based on [51, 66-67], NWA = Northwestern Atlantic, SWA = Southwestern Atlantic, MAR = Mid-Atlantic Ridge, NEA = Northeast Atlantic, TEA = Tropical Eastern Atlantic. New = new record for Gabon.

|  |  |  |  |  |  | TEA | | | | |
| --- | --- | --- | --- | --- | --- | --- | --- | --- | --- | --- |
| Family | Taxa | NWA | SWA | MAR | NEA | Cabo Verde | Sao Tome | NW Africa | Tropical West Africa | Gabon |
| Carcharhinidae | *Carcharhinus falciformis* | X | X | X | X | X | X | X | X |  |
| Dasyatidae | *Dasyatis margarita* |  |  |  |  | X |  |  | X |  |
| Muraenidae | *Gymnothorax afer* |  |  |  |  | X | X | X | X |  |
| Muraenidae | *Muraena melanotis* |  | X |  |  | X | X | X | X |  |
| Synodontidae | *Synodus synodus* | X | X | X | X | X | X |  |  | New |
| Holocentridae | *Myripristis jacobus* | X | X | X | X | X | X |  | X |  |
| Serranidae | *Cephalopholis nigri* |  |  |  | X | X | X |  | X |  |
| Serranidae | *Cephalopholis taeniops* |  |  |  |  | X | X | X | X |  |
| Serranidae | *Epinephelus adscensionis* | X | X | X |  | X | X |  | X | New |
| Serranidae | *Epinephelus aeneus* |  |  |  | X | X | X | X | X |  |
| Serranidae | *Epinephelus itajara* | X | X |  | X | X | X | X | X |  |
| Serranidae | *Paranthias furcifer* | X | X | X |  |  | X |  |  | New |
| Serranidae | *Serranus accraensis* |  |  |  |  |  |  |  | X |  |
| Priacanthidae | *Priacanthus arenatus* | X | X |  | X | X | X | X | X |  |
| Cirrhitidae | *Cirrhitus atlanticus* |  |  |  |  |  | X | X | X | New |
| Apogonidae | *Apogon imberbis* |  |  |  | X | X | X | X | X | New |
| Echeneidae | *Remora brachyptera* | X | X | X | X | X | X | X | X |  |
| Echeneidae | *Remora remora* | X | X | X | X | X | X | X | X |  |
| Rachycentridae | *Rachycentron canadum* | X | X | X | X | X | X | X | X |  |
| Carangidae | *Carangoides bartholomaei* | X | X |  |  |  | X |  |  | New |
| Carangidae | *Caranx crysos* | X | X | X | X | X | X |  | X |  |

Appendix II continued.

|  |  |  |  |  |  | TEA | | | | |
| --- | --- | --- | --- | --- | --- | --- | --- | --- | --- | --- |
| Family | Taxa | NWA | SWA | MAR | NEA | Cabo Verde | Sao Tome | NW Africa | Trop. W Africa | Gabon |
| Carangidae | *Caranx fisheri* |  |  | X | X |  | X | X | X |  |
| Carangidae | *Caranx hippos* | X | X |  |  | X | X | X | X |  |
| Carangidae | *Caranx latus* | X | X | X | X | X | X |  | X | New |
| Carangidae | *Elagatis bipinnulata* | X | X | X | X | X | X | X | X |  |
| Carangidae | *Seriola rivoliana* | X | X | X | X | X |  |  |  | New |
| Carangidae | *Trachinotus goreensis* |  |  |  |  | X | X | X | X |  |
| Carangidae | *Trachinotus ovatus* |  |  | X | X | X | X | X | X |  |
| Lutjanidae | *Lutjanus agennes* |  |  |  |  | X | X |  | X |  |
| Lutjanidae | *Lutjanus dentatus* |  |  |  |  |  | X | X | X |  |
| Lutjanidae | *Lutjanus endecacanthus* |  |  |  |  | X | X | X | X |  |
| Lutjanidae | *Lutjanus goreensis* |  |  |  | X | X | X | X | X |  |
| Haemulidae | *Plectorhinchus macrolepis* |  |  |  | X | X | X | X | X |  |
| Haemulidae | *Pomadasys incisus* |  |  |  | X | X | X | X | X |  |
| Haemulidae | *Pomadasys jubelini* |  |  |  |  | X |  | X | X |  |
| Centracanthidae | *Spicara melanurus* |  |  |  |  | X | X | X | X | New |
| Sciaenidae | *Pseudotolithus senegalensis* |  |  |  |  |  |  |  | X |  |
| Mullidae | *Pseudupeneus prayensis* |  |  |  | X | X | X | X | X |  |
| Kyphosidae | *Kyphosus incisor/sectatrix* | X | X | X | X | X | X | X | X |  |
| Chaetodontidae | *Chaetodon hoefleri* |  |  |  | X | X | X | X | X |  |
| Chaetodontidae | *Chaetodon robustus* |  |  |  |  | X | X | X | X | New |
| Chaetodontidae | *Prognathodes marcellae* |  |  |  | X | X | X | X | X |  |
| Pomacanthidae | *Holacanthus africanus* |  |  |  |  | X | X | X | X |  |
| Pomacentridae | *Abudefduf hoefleri* |  |  |  |  | X | X |  | X | New |
| Pomacentridae | *Abudefduf saxatilis* | X | X | X | X | X | X | X | X |  |
| Pomacentridae | *Chromis multilineata* | X | X | X |  | X | X |  | X | New |

Appendix II continued.

|  |  |  |  |  |  | TEA | | | | |
| --- | --- | --- | --- | --- | --- | --- | --- | --- | --- | --- |
| Family | Taxa | NWA | SWA | MAR | NEA | Cabo Verde | Sao Tome | NW Africa | Trop. W Africa | Gabon |
| Pomacentridae | *Microspathodon frontatus* |  |  |  |  |  | X |  | X | New |
| Pomacentridae | *Stegastes imbricatus* |  |  |  |  | X | X | X | X |  |
| Labridae | *Bodianus pulchellus* | X | X |  |  |  | X |  |  | New |
| Labridae | *Bodianus speciosus* |  |  |  |  | X | X | X | X |  |
| Labridae | *Thalassoma newtoni* |  |  |  |  |  | X |  | X | New |
| Scaridae | *Scarus hoefleri* | X | X |  | X | X | X | X | X |  |
| Blenniidae | *Ophioblennius atlanticus* | X | X | X |  | X | X | X | X | New |
| Blenniidae | *Parablennius goreensis* |  |  |  |  |  |  | X | X | New |
| Ephippidae | *Chaetodipterus lippei* |  |  |  |  | X |  |  | X |  |
| Acanthuridae | *Acanthurus monroviae* |  | X |  | X | X | X | X | X |  |
| Scombridae | *Auxis rochei* | X | X | X | X | X | X | X | X |  |
| Sphyraenidae | *Sphyraena barracuda* | X | X | X | X | X | X | X | X |  |
| Sphyraenidae | *Sphyraena guachancho* | X | X | X | X | X |  |  | X | New |
| Balistadae | *Balistes capriscus* |  |  |  | X | X | X | X | X |  |
| Balistadae | *Balistes punctatus* | X | X |  | X | X | X | X | X |  |
| Monacanthidae | *Cantherhines pullus* | X | X |  |  |  | X | X | X | New |
| Tertaodontidae | *Canthigaster supramacula* |  |  |  |  | X | X |  | X | New |
| Tetraodontidae | *Sphoeroides marmoratus* |  |  |  | X | X | X | X | X | New |
| Diodontidae | *Diodon holocanthus* | X | X | X | X | X | X |  | X | New |
